# Changes in Intraocular Pressure and Ganglion Cell Complex Thickness After Pars Plana Vitrectomy in Patients With and Without Diabetes Mellitus

**DOI:** 10.7759/cureus.107201

**Published:** 2026-04-16

**Authors:** Srinidhi N, Gayatree Mohanty, Manmath K Das, Soumyakanta Mohanty, Aanchal Singhal, Manisha Choudhary

**Affiliations:** 1 Ophthalmology, Kalinga Institute of Medical Sciences, Bhubaneswar, IND

**Keywords:** diabetes mellitus, ganglion cell complex, intraocular pressure, optical coherence tomography, pars plana vitrectomy (ppv), retinal neurodegeneration

## Abstract

Introduction

Pars plana vitrectomy (PPV) is widely performed for various vitreoretinal disorders. However, postoperative elevation of intraocular pressure (IOP) and thinning of the ganglion cell complex (GCC) may adversely affect visual outcomes, particularly in diabetic patients. These changes may be attributed to altered ocular biomechanics, inflammation, and compromised microvascular perfusion in diabetes.

Objective

The objective of this study was to evaluate postoperative changes in IOP and GCC thickness following PPV and to compare outcomes between diabetic and non-diabetic patients. The study also aims to identify early postoperative trends that may predict long-term neuroretinal damage.

Methods

This prospective cohort study included 72 patients (36 diabetics and 36 non-diabetics) undergoing PPV for various vitreoretinal indications. IOP and GCC thickness were measured preoperatively and postoperatively on Day 1, Day 7, and at 1 month using Goldmann applanation tonometry and spectral-domain optical coherence tomography (SD-OCT). Patients were followed up at regular intervals, and any postoperative complications were documented. Statistical analysis was performed using SPSS, with p < 0.05 considered statistically significant.

Results

Baseline demographic and ocular parameters were comparable between the groups. Postoperative IOP was similar on Day 1 but was significantly higher in diabetic patients at Day 7 (p = 0.020) and at 1 month (p = 0.002). IOP spikes were observed only in the diabetic group, suggesting a higher susceptibility to pressure dysregulation. GCC thinning was significantly greater in diabetics at 1 month (p = 0.007), whereas non-diabetic patients showed minimal change. A trend toward progressive GCC reduction was noted in diabetics over time, indicating possible ongoing neurodegenerative changes.

Conclusion

Diabetes mellitus is associated with sustained postoperative IOP elevation and significant GCC thinning following PPV. These findings highlight the need for closer postoperative monitoring and early intervention in diabetic patients to prevent neuroretinal damage and optimize visual outcomes. Prophylactic IOP control and timely management strategies may play a crucial role in improving surgical outcomes in this high-risk group.

## Introduction

Pars plana vitrectomy (PPV) is a widely performed vitreoretinal procedure indicated for conditions such as proliferative diabetic retinopathy, epiretinal membranes, macular holes, vitreous hemorrhage, and retinal detachment [1,2]. Despite significant advances in surgical techniques and instrumentation, postoperative complications such as intraocular pressure (IOP) elevation and ganglion cell complex (GCC) thinning remain important determinants of visual prognosis.

IOP elevation after PPV may be transient or sustained, occasionally leading to secondary glaucoma [3-5]. Early postoperative spikes are commonly attributed to inflammation, retained viscoelastic material, or expansile gas tamponade, whereas long-term elevation may result from oxidative stress, trabecular meshwork dysfunction, or silicone oil emulsification [6,7]. The absence of the vitreous body increases intraocular oxygen diffusion, promoting free radical formation and subsequent impairment of aqueous outflow [6].

Patients with diabetes mellitus (DM) may be particularly susceptible to such changes due to underlying microvascular compromise, impaired autoregulation, and pre-existing retinal neurodegeneration [8-10]. Optical coherence tomography (OCT) studies have demonstrated thinning of the ganglion cell-inner plexiform layer even in diabetic individuals without clinically evident retinopathy, suggesting reduced neuronal reserve [9,10].

In addition to IOP changes, PPV itself may contribute to structural alterations in the GCC. Greater thinning has been reported, particularly in cases involving internal limiting membrane peeling, possibly due to mechanical trauma and Müller cell dysfunction [11]. Diabetes-associated oxidative stress and impaired antioxidant mechanisms may further accelerate ganglion cell loss in these patients [6,8].

Monitoring IOP and GCC thickness postoperatively is essential for early detection of complications. While IOP measurement remains standard clinical practice, OCT provides a reliable and reproducible method for detecting subtle neurostructural changes [12-14]. However, existing literature shows variability in reported outcomes, particularly when comparing diabetic and non-diabetic patients.

This study aims to evaluate changes in IOP and GCC thickness following PPV in patients with and without diabetes mellitus, addressing an important gap in current evidence and improving risk stratification for postoperative visual outcomes.

## Materials and methods

Study design and setting

This was a prospective cohort study conducted in the Department of Ophthalmology at Pradyumna Bal Memorial Hospital, Kalinga Institute of Medical Sciences (KIMS), Bhubaneswar, Odisha, India, from February 2024 to February 2026. Patients undergoing PPV for various vitreoretinal conditions were enrolled and followed longitudinally to assess changes in IOP and GCC thickness. A prospective cohort design enabled comparison between patients with and without diabetes while ensuring standardized, prospective data collection. The study was approved by the Institutional Ethics Committee, Kalinga Institute of Medical Sciences (approval number: KIIT/KIMS/IEC/1517/2024), and adhered to the Declaration of Helsinki.

Study population and sample size

Inclusion and Exclusion Criteria

Individuals aged 18 years or older undergoing primary PPV for indications such as vitreous haemorrhage, tractional retinal detachment, epiretinal membrane, or macular hole were included in the study. Patients with and without diabetes were both considered, provided they gave informed written consent for participation. Patients with pre-existing glaucoma or ocular hypertension, optic nerve pathologies (such as optic atrophy or compressive neuropathy), or dense corneal opacity interfering with applanation tonometry or OCT were excluded. Additionally, those with a history of intraocular surgery other than cataract extraction, intraoperative complications (e.g., scleral perforation, suprachoroidal haemorrhage, or conversion to scleral buckling), or inability to comply with follow-up visits were also excluded.

Sampling and Sample Size

Purposive sampling was used, and consecutive eligible patients were recruited until the required sample size was achieved. Based on prior studies, a minimum of 72 participants was calculated (95% power, 5% significance), accounting for potential dropouts.

Study groups and parameters

Participants were divided into two groups: diabetic (Group A) and non-diabetic (Group B). Data collected included demographic details, best-corrected visual acuity, slit-lamp and fundus findings, and intraoperative variables. IOP was measured using Goldmann applanation tonometry, and GCC thickness was assessed using spectral-domain optical coherence tomography (SD-OCT) at baseline, day 1, day 7, and at one month postoperatively.

Study procedure and data collection

All patients underwent standard 23- or 25-gauge PPV performed by experienced surgeons. Additional procedures, such as internal limiting membrane peeling and tamponade use, were performed as indicated. Postoperative evaluations were conducted at day 1, day 7, and one month. Data were recorded in structured forms, with OCT measurements independently reviewed to minimize bias.

Statistical analysis 

Data were analyzed using IBM SPSS Statistics for Windows, version 25.0 (IBM Corp., Armonk, New York, United States). Continuous variables were expressed as mean ± standard deviation (SD), while categorical variables were presented as frequencies and percentages. Comparisons between diabetic and non-diabetic groups were performed using the independent samples t-test (two-tailed) for continuous variables and the chi-square test for categorical variables. A p-value of <0.05 was considered statistically significant.

## Results

Table 1 presents the demographic profile of the study participants, with equal distribution between the diabetic and non-diabetic groups (n = 36 each). The mean age was comparable between the groups (57.03 ± 11.74 vs 58.36 ± 8.44 years), with no statistically significant difference (t = 0.55, p = 0.582). Similarly, gender distribution showed no significant difference between the groups (χ² = 1.41, p = 0.234), despite a slightly higher proportion of females in the non-diabetic group. Overall, both groups were demographically comparable, minimizing the influence of age and gender as potential confounding factors.

**Table 1:** Demographic profile of study participants (N = 72) independent samples t-test used for continuous variables; Chi-square test used for categorical variables.

Parameter	Diabetic (n = 36), mean±SD	Non-Diabetic (n = 36), mean±SD	Test Statistic	p-value
Age (years)	57.03 ± 11.74	58.36 ± 8.44	t = 0.55	0.582
Gender (Male/Female)	18/18	13/23	χ² = 1.41	0.234

Table 2 presents the baseline comparison of preoperative IOP and GCC thickness between the two groups. The mean preoperative IOP was significantly higher in the diabetic group (13.83 ± 1.46 mmHg) compared to the non-diabetic group (11.72 ± 1.37 mmHg), and this difference was statistically significant (t = 6.32, p < 0.001). In contrast, the mean GCC thickness was comparable between the two groups (93.97 ± 3.74 µm in diabetics vs 94.17 ± 8.01 µm in non-diabetics), with no statistically significant difference observed (t = −0.14, p = 0.439). Overall, while baseline IOP differed significantly between groups, retinal structural status as assessed by GCC thickness was similar preoperatively.

**Table 2:** Baseline comparison of preoperative IOP and GCC thickness between the two groups Independent samples t-test used IOP: intraocular pressure; GCC: ganglion cell complex

Parameter	Diabetic (n = 36), mean ± SD	Non-Diabetic (n = 36), mean ± SD	Test Statistic	p-value
Preoperative IOP (mmHg)	13.83 ± 1.46	11.72 ± 1.37	t = 6.32	<0.001
Preoperative GCC thickness (µm)	93.97 ± 3.74	94.17 ± 8.01	t = −0.14	0.439

Table 3 compares postoperative IOP between the diabetic and non-diabetic groups at different follow-up intervals. On postoperative day 1, the mean IOP was identical in both groups (16.89 mmHg), with no statistically significant difference (t = 0.00, p = 1.00). On day 7, the diabetic group demonstrated significantly higher IOP (17.11 ± 3.33 mmHg) compared to the non-diabetic group (15.17 ± 2.64 mmHg), with a mean difference of 1.94 mmHg (t = 2.38, p = 0.020). At one month, this difference remained significant, with higher IOP in diabetics (16.56 ± 3.63 mmHg) than non-diabetics (14.72 ± 2.49 mmHg), showing a mean difference of 1.84 mmHg (t = 3.20, p = 0.002). Overall, while immediate postoperative IOP was comparable, patients with diabetes exhibited significantly higher IOP during subsequent follow-up.

**Table 3:** Postoperative IOP comparison between the two groups Independent samples t-test was used IOP: intraocular pressure; SD: standard deviation

Parameter	Diabetic (n = 36), mean ± SD (mmHg)	Non-Diabetic (n = 36), mean ± SD	Mean IOP difference	Test Statistic	p-value
IOP on Day 1 (mmHg)	16.89±3.04	16.89±2.21	0.00 mmHg	t = 0.00	1.00
IOP on Day 7 (mmHg)	17.11 ± 3.33	15.17 ± 2.64	1.94 mmHg	t = 2.38	0.020
IOP at 1 Month (mmHg)	16.56 ± 3.63	14.72 ± 2.49	1.84 mmHg	t = 3.20	0.002

Table 4 compares postoperative GCC thickness between diabetic and non-diabetic groups across follow-up intervals. On postoperative day 1, GCC thickness was comparable between the groups (95.06 ± 3.66 µm vs 96.08 ± 8.07 µm), with no statistically significant difference (t = −0.71, p = 0.48). On day 7, a greater reduction in GCC thickness was observed in the diabetic group (93.11 ± 3.83 µm) compared to the non-diabetic group (95.61 ± 8.70 µm); however, this difference did not reach statistical significance (t = −1.61, p = 0.11). At one month, GCC thickness was significantly lower in the diabetic group (90.58 ± 3.61 µm) compared to the non-diabetic group (94.97 ± 8.77 µm), with a mean difference of 4.39 µm (t = −2.78, p = 0.007). Overall, while early postoperative changes were comparable, patients with diabetes demonstrated significantly greater GCC thinning at one month.

**Table 4:** Postoperative GCC thickness comparison between the two groups Independent samples t-test was used GCC: ganglion cell complex thickness; SD: standard deviation

Parameter	Diabetic (n = 36), mean ± SD	Non-Diabetic (n = 36), mean ± SD	Mean GCC difference	Test statistic	p-value
GCC on Day 1 (µm)	95.06 ± 3.66	96.08 ± 8.07	1.02	t = -0.71	0.48
GCC on Day 7 (µm)	93.11 ± 3.83	95.61 ± 8.70	2.50	t = −1.61	0.11
GCC at 1 Month (µm)	90.58 ± 3.61	94.97 ± 8.77	4.39	t = −2.78	0.007

## Discussion

The present study evaluated postoperative changes in IOP and GCC thickness following PPV, with a comparative analysis between patients with and without diabetes. The findings demonstrate that diabetes mellitus significantly influences postoperative ocular physiology, particularly in terms of sustained IOP elevation and progressive neuroretinal thinning.

Baseline demographic parameters, including age and gender, were comparable between groups, minimizing potential confounding effects. Preoperative IOP was significantly higher in patients with diabetes, which should be considered when interpreting postoperative IOP outcomes; however, baseline GCC thickness was comparable between groups, indicating similar preoperative retinal structural status.

Postoperatively, both groups exhibited an initial rise in IOP on Day 1, consistent with known early postoperative responses following PPV [15-17]. However, a key finding was the divergence in IOP trends thereafter. Patients with diabetes demonstrated significantly higher IOP on Day 7 and at one month compared to patients without diabetes, suggesting impaired pressure regulation or prolonged inflammatory response. Furthermore, elevated IOP occurred exclusively in the diabetic group, affecting over one-quarter of patients on Day 1 and persisting in a subset at later time points. In contrast, patients without diabetes showed a gradual normalization of IOP without clinically significant spikes.

Structural outcomes also differed between groups. Although baseline GCC thickness was comparable, diabetic patients exhibited progressive postoperative thinning, reaching statistical significance at one month. This finding indicates increased vulnerability of retinal ganglion cells in diabetic eyes. In contrast, patients without diabetes showed minimal and non-significant GCC changes, suggesting relative structural preservation.

The association between higher postoperative IOP and greater GCC thinning in diabetics highlights a potential mechanistic link between pressure-related stress and neuroretinal damage. Given that diabetes is associated with microvascular compromise and neurodegeneration, these findings suggest that surgical stress and postoperative IOP fluctuations may further exacerbate neuronal loss in this population [18,19].

The study has several strengths, including a balanced comparative design, standardized follow-up at multiple early postoperative time points, and simultaneous assessment of both functional (IOP) and structural (GCC) parameters. The inclusion of categorical IOP outcomes enhances clinical relevance by identifying patients at risk for ocular hypertension.

Clinically, these findings underscore the importance of extended postoperative monitoring in diabetic patients undergoing PPV. While immediate postoperative IOP may appear comparable, delayed elevation is more likely in diabetics, necessitating vigilant follow-up beyond the first postoperative day. Additionally, the incorporation of OCT-based GCC assessment may facilitate early detection of neuroretinal compromise.

Future studies should include longer follow-up durations to determine whether these early changes persist or progress to clinically significant glaucomatous damage. Further stratification based on glycemic control, duration of diabetes, and presence of diabetic retinopathy may help identify high-risk subgroups. Evaluation of surgical variables and preventive strategies, including prophylactic IOP-lowering therapy, may also provide insights into optimizing postoperative outcomes.

This study has several limitations, such as a relatively small sample size (n=72), which may limit generalizability and statistical power. The follow-up period was short (one month), restricting assessment of long-term outcomes such as persistent IOP elevation or GCC changes. Important diabetes-related factors (duration, HbA1c, and diabetic retinopathy severity) and lens status were not stratified, potentially influencing results. Surgical variables (tamponade type, gauge, laser use, and steroid therapy) were not individually analysed. Functional outcomes such as best corrected visual acuity and visual fields were not assessed. Additionally, OCT-based GCC measurements may be affected by device variability and segmentation errors

## Conclusions

This study demonstrates that diabetes mellitus significantly influences postoperative outcomes following PPV. Patients with diabetes exhibited a higher risk of sustained IOP elevation and a greater frequency of postoperative IOP spikes compared to patients without diabetes. In addition, the former showed significant progressive thinning of the GCC within the first postoperative month, indicating increased neuroretinal vulnerability. The findings highlight a potential association between postoperative IOP elevation and GCC thinning, suggesting that pressure-related stress may contribute to early neuroretinal damage in diabetic eyes.

These results emphasize the importance of individualized postoperative care in diabetic patients. Close and prolonged IOP monitoring, along with early initiation of IOP-lowering therapy when required, is essential to prevent pressure-related complications. Incorporating OCT-based GCC assessment into routine follow-up may aid in early detection of subclinical neuroretinal damage. Overall, patients with diabetes undergoing PPV should be considered a high-risk group, requiring enhanced surveillance and targeted management strategies to optimize structural and functional visual outcomes.
